# Supplementary material for: Continuous versus Intermittent Enteral Tube Feeding for Critically Ill Patients: A Prospective, Randomized Controlled Trial
Source: Nutrients. 2022 Feb 4;14(3):664. doi: 10.3390/nu14030664 (PMC8839656; doi:10.3390/nu14030664)
Supplement: Supplementary file 1 [file nutrients-14-00664-s001.zip › nutrients-1582667-supplementary.pdf]

a

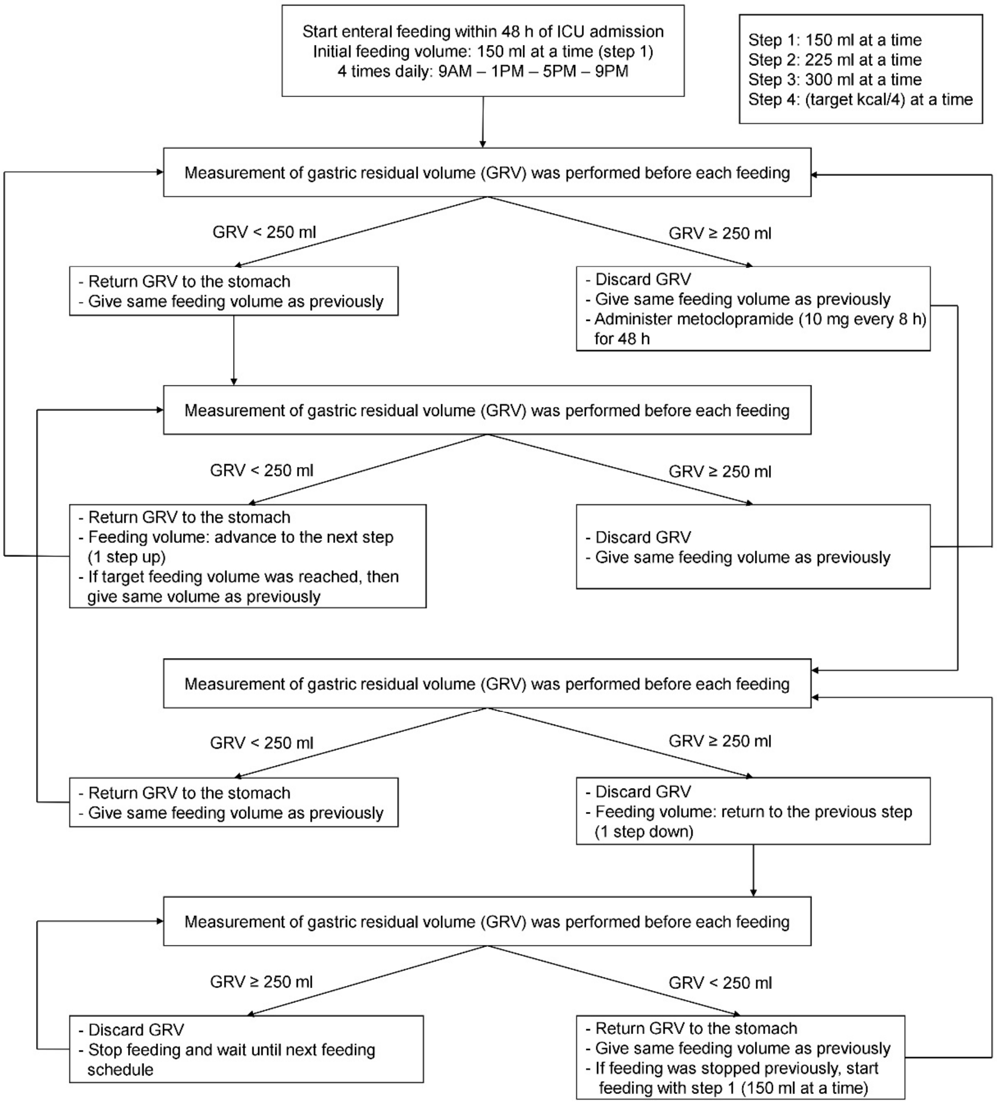
Start enteral feeding within 48 h of ICU admission
Initial feeding volume: 150 ml at a time (step 1)
4 times daily: 9AM – 1PM – 5PM – 9PM
Step 1: 150 ml at a time
Step 2: 225 ml at a time
Step 3: 300 ml at a time
Step 4: (target kcal/4) at a time
Measurement of gastric residual volume (GRV) was performed before each feeding
GRV < 250 ml
GRV ≥ 250 ml
- Return GRV to the stomach
- Give same feeding volume as previously
- Discard GRV
- Give same feeding volume as previously
- Administer metoclopramide (10 mg every 8 h) for 48 h
Measurement of gastric residual volume (GRV) was performed before each feeding
GRV < 250 ml
GRV ≥ 250 ml
- Return GRV to the stomach
- Feeding volume: advance to the next step (1 step up)
- If target feeding volume was reached, then give same volume as previously
- Discard GRV
- Give same feeding volume as previously
Measurement of gastric residual volume (GRV) was performed before each feeding
GRV < 250 ml
GRV ≥ 250 ml
- Return GRV to the stomach
- Give same feeding volume as previously
- Discard GRV
- Feeding volume: return to the previous step (1 step down)
Measurement of gastric residual volume (GRV) was performed before each feeding
GRV ≥ 250 ml
GRV < 250 ml
- Discard GRV
- Stop feeding and wait until next feeding schedule
- Return GRV to the stomach
- Give same feeding volume as previously
- If feeding was stopped previously, start feeding with step 1 (150 ml at a time)

b

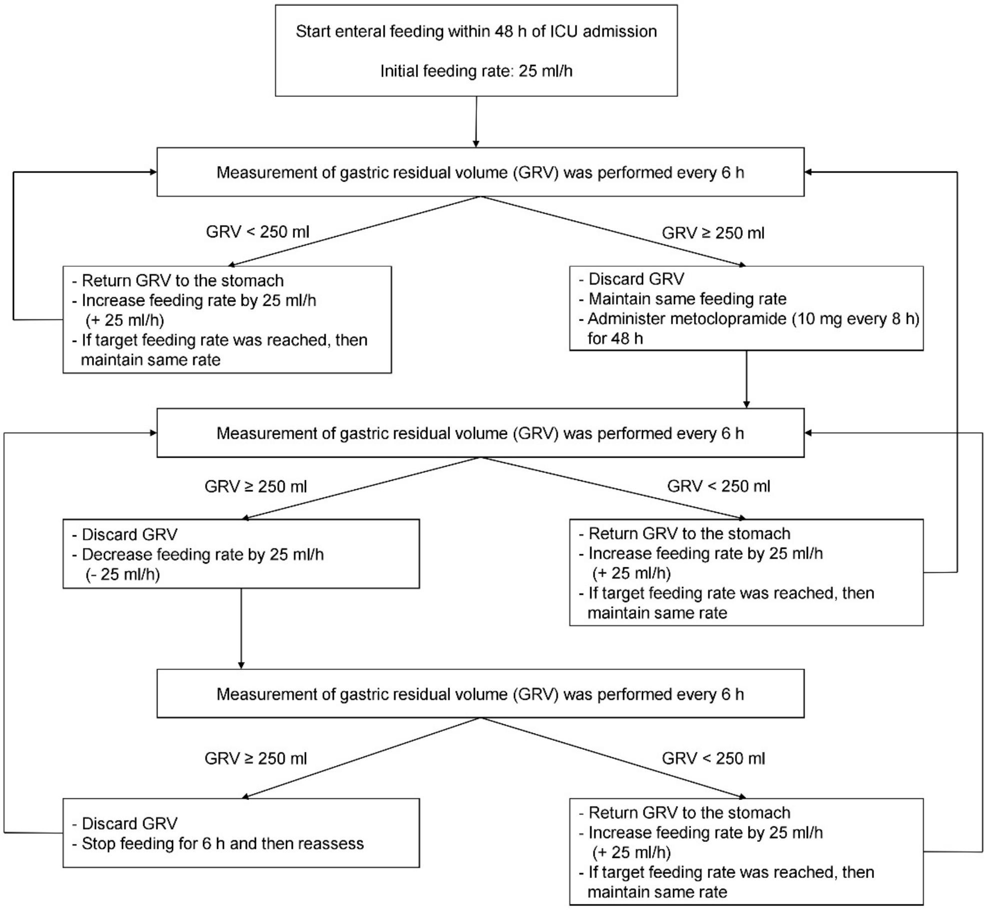

Figure S1. Detailed enteral feeding algorithm for the intermittent enteral feeding group (a) and continuous enteral feeding group (b). ICU = intensive care units.
